# Combined Effect of Chia Flour and Soy Lecithin Incorporation on Nutritional and Technological Quality of Fresh Bread and during Staling

**DOI:** 10.3390/foods9040446

**Published:** 2020-04-07

**Authors:** Imen Bel Hadj Ahmed, Ahmed Hannachi, Claudia Monika Haros

**Affiliations:** 1 Institute of Agrochemistry and Food Technology (IATA-CSIC), Av. Agustín Escardino 7 Parque Científico, 46980 Paterna-Valencia, Spain; imenbelhadjahmed@yahoo.fr; 2 GPSI, National Engineering School of Gabes, University of Gabes, Omar Ibn El Khattab Street, Gabes 6029, Tunisia; ahmed.hannachi@enig.rnu.tn

**Keywords:** bread staling, chia, soy lecithin, techno-functional properties, physicochemical characteristics

## Abstract

The objectives of the present investigation are to study the interaction and optimize the blend composition of flour of grinded Chia seeds, combined to Soy lecithin, a bread making improver, in a way to enhance the nutritional/functional value of bread without impairing its technological quality and to delay its staling rate. Nine formulations were prepared following a Central Composite Design. Technological attributes were evaluated both for fresh and stored bread. In the Response Surface Methodology (RSM) a desirability function identified the optimum doses of chia and lecithin incorporation to obtain the highest specific volume and the lowest crumb firmness. Compared to the control, samples with chia and lecithin significantly increased the nutritional value of bread. An innovative and interesting synergy was found in lecithin/chia combination to enhance the specific volume, to reduce the initial crumb firmness and to delay bread staling by retarding crumb firmness and reducing its water loss during storage. Using the RSM, the optimum blend containing (4.04%-Chia/1%-Lecithin) showed fresh bread with maximum specific volume and minimum crumb firmness. Whereas, bread combining the optimum blend (3.43%-Chia/1%-Lecithin) and stored for two days at room temperature showed the minimum crumb firmness.

## 1. Introduction

Worldwide, consumption of soft wheat makes it a primordial cereal which occupies an important position in the international nutrition. In raw state, this cereal is the source of a large amount of nutrients required by the human organism. However, processing, which is prerequisite for manufacturing attractive products and obtaining a food in a suitable form and good palatability, may decrease the levels of the bioactive components in grains and also alter the bioavailability of these components [1]. This is the case for the bread making process from wheat flour, in which the extraction rate, the milling and the baking and storage conditions are among the factors that damage the nutritional value and the technological quality of fresh bread. Besides, this product has a short shelf-life and during its storage a number of chemical and physical alterations occur, known as staling [2].

Starch retrogradation, one of the major factors of staling, includes the short-term development of amylose gel network structure (crystallization) and a long-term reordering of amylopectin, which is a much slower process involving the outer branches recrystallization of this polymer [3,4]. Staling is also affected by the distribution of water between starch and gluten and, as a result, the crumb will become increasingly firm with time [5].

Today, several anti-staling agents used in the bread making industry provide the opportunity to delay its quality properties degradation. Soy lecithin is an anti-staling agent which has an influence on starch retrogradation which is the key factor contributing to staling. This emulsifier acts by the formation of inclusion complexes with amylose or the outer branches of the amylopectin molecule, and delaying the swelling of starch granules [6]. On the other side, many attempts have been made to increase the nutritional value of bread by incorporating dietary fibers in the formulations [7,8]. One grain with interesting properties for this capacity is chia seeds (*Salvia hispanica* L). Chia is a summer annual plant belonging to the Labiatae family [9]. Now that numerous studies have shown the remarkable nutritional properties of the chia seed, it is recommended for consumption because of its high oil, protein, antioxidants, minerals and dietary fiber contents [10].

Due to the fact that the quality of a bread product mainly lies in the content and quality of the proteins that make up gluten, the substitution of wheat flour with other ingredients, could alter its cohesive and viscoelastic properties during the kneading. Consequently, the resulting dough loses its ability to retain gas during fermentation, meaning the bread produced is denser, with lower volume and a more compact crumb structure [11]. These results urge us to find solutions to compensate for the poor wheat flour gluten network. In view of these above findings, the goals of this study are, first, to investigate the interaction between the addition of grounded chia seeds (*Salvia hispanica* L) combined with soy lecithin to wheat flour bread, second, to optimize the blend composition in a way to enhance the nutritional value of wheat flour bread, without impairing its technological quality.

## 2. Materials and Methods 

### 2.1. Raw Material

Commercial Spanish wheat flour (4.1 ± 0.2 g/100 g lipids, 0.53 ± 0.01 g/100 g ash, 11.6 ± 0.4 g/100 g protein, 0.15 ± 0.05 g/100 g dietary fibers 83.3 ± 0.3 g/100 g carbohydrates), was purchased from the local market. The flour alveograph parameters were tenacity, P: 60 ± 1.5 mm; extensibility, L: 62.6 ± 2.8 mm; and deformation work, W: 148.6 ± 2.3 10^−4^ J. Chia seeds (36.1 ± 0.4 g/100 g lipids; 18.51 ± 0.3 g/100 g proteins; 30.41 ± 0.4 g/100 g total dietary fiber; 2.11 ± 0.02 g/100 g ash) were kindly granted by Primaria Premium Raw Materials S.L. (Valencia, Spain). Soy lecithin was provided by SIPA (Tunis, Tunisia). Compressed yeast (*Saccharomyces cerevisiae*, Levamax, Spain) was used as a starter for the bread-making process.

### 2.2. Experimental Design 

Chia seeds flour and soy lecithin amounts (the two independent variables) were added to the formulation according to a 2^2^ central composite rotational design (CCRD) of a Response Surface Methodology (RSM). The quantities added ranged from 0 to 10% on flour basis of chia and from 0 to 1% on flour basis of Soy lecithin. Nine assays were conducted including four factorial points (2^2^) and five repetitions of the central point (Table 1). The study was designed with the help of MiniTab 17 software, 2007.

### 2.3. Proximal Composition

Proximal composition was determined in triplicate for fresh bread derived from each formulation. Protein determination was carried out by the Kjeldahl technique. Lipid content was extracted with petroleum ether under reflux conditions by the Soxhlet technique [12], whereas ash content was determined by incineration in a muffle at 910 °C. The dietary fiber content was measured by the total dietary fiber assay procedure [13].

### 2.4. Dough Mixing Properties Determination 

To evaluate the impact of the incorporation of chia and lecithin on the mixing properties for each formulation, a Farinograph (Brabender, Duisburg, Germany) was used, following the official standard method with slight modifications [12].

### 2.5. Breadmaking Procedure

The bread control dough formula consisted of: wheat flour (500 g), compressed yeast (2.5% flour basis), sodium salt (1.6% flour basis), and distiller water (up to optimum absorption, 500 Brabender Units, BU). The ingredients were mixed for 4.0 min, rested for 10 min, divided (100 g), kneaded and then rested (15 min). Doughs were manually sheeted and rolled, proofed during 1 h (at 28 °C and 85% relative humidity) and baked at 220 °C/25 min. The chia seeds were added at 5% and 10% on the flour basis and the soy lecithin at 0.5 and 1.0% on flour basis according to the formulations described in Table 1. Fermentation was monitored by measuring the temperature and volume increase of the dough at regular time intervals. After the fermentation step, the doughs were baked in an electric oven and cooled at room temperature for 75 min, for subsequent analysis.

### 2.6. Technological Evaluation 

Bread quality was assessed for fresh and stored bread. Bread loaves were stored in polyethylene bags at 25 °C for four days. All the experiments of this part were done in triplicate.

Bread volume was determined by a rapeseed displacement method and the average specific volume (volume/weight) was calculated. Moisture content evaluation was carried out both for the whole bread loaves and the bread crumbs, according to the American Association of Cereal Chemists (AACC) Method [12].

Texture profile analysis was carried out using the Texture Analyzer (Stable Micro Systems, Godalming, United Kingdom). Two 25-mm thick slices were compressed in the center of the Texture Analyzer platform using a cylindrical probe of 36 mm in diameter under the following conditions: speed of 1.7 mm/s for the test; speed of 10 mm/s for the post-test; 40% compression and 5 g trigger force. Firmness, springiness, cohesiveness and chewiness were recorded [12]. 

### 2.7. Data analyses and Validation of RSM Results

Different models were studied to fit the experimental data using a MiniTab 17 software. The analysis of variance (ANOVA) was used to evaluate the significance of each equation (*p* < 0.05). In every case, the quadratic model was the most adequate accurate model. Specific volume and crumb firmness are the most critical attributes of bread. RSM was used to optimize the composition of chia and lecithin blends. A numerical desirability function was used to find the optimal composition in order to obtain bread with maximum specific volume and minimum crumb firmness values. Experimental data were fitted to the quadratic model:

y = b_0_ + b_1_X_1_ + b_2_X_2_ + b_3_X_1_^2^ + b_4_X_2_^2^+ b_5_X_1_X_2_ (1)

where b_0_ is a constant; b_1_ and b_2_ express linear effects of each variable; b_3_ and b_4_ are square coefficients; and b_5_ shows the interaction between the variables. Response surfaces of the models were plotted as a function of the two variables.

## 3. Results and Discussion

### 3.1. Effect of Chia Flour and Soya Lecithin Addition on the Dough Mixing Properties 

The results of the mixing properties are summarized in Table 2. The enriched samples showed a significantly higher water absorption compared to the control bread. The absorption capacity increased ~13% for the two formulations with 10% of chia; and between 2% and 4% in cases of Ch0-Lec1 and Ch5-Lec0.5, respectively. 

The hydration increase is due to lecithin and especially chia substitution. A higher water absorption was observed in mixtures containing the biggest amount of Chia. According to Segura-Campos et al. [14], extracted polysaccharides from whole chia flour are able to absorb water in a high proportion. In line with this, the studies into chia mucilage by Muñoz et al. [15] reported that it is able to hydrate 27 times its own weight. The arrival time parameter was not significantly affected by the chia and lecithin incorporation in all the formulations, although there is a tendency to increase with the addition of chia flour.

During dough development, it reaches a maximum consistency and henceforth it is able to resist to deformation for some time, which determines the dough’s stability.

Stability, development time and breakdown time parameters are increased with chia increase or chia-lecithin combination. This finding could mainly be due to the lower rate of hydration of the components due to an increased competition for water between chia fiber and gluten proteins. The study of Steffolani et al. [11] may confirm this interpretation. In fact, they found that hydration of chia prior to the incorporation to the dough, induces a decrease of development and stability times, as compared to the control. They reported that the water absorption by the mucilage had already occurred and that there was no direct competition with the gluten for water, thus facilitating a more rapid development and stability. However, the sample with exclusive substitution of lecithin Ch0-Lec1 showed no significant effect on these parameters, compared to control sample. 

### 3.2. Proximal Composition 

The bread samples’ proximal composition are presented in Table 3. As was expected, soy lecithin had no significant effect on bread nutritional value. Changes in chemical composition of tested blends were clearly attributed to chia addition. A high content of protein, ash, and dietary fiber in chia flour contributed to a significant increase of the content of these components in the resulted breads. This behavior was also found by Pizzaro et al. [16] in a cake formulation with chia at 15% substitution, and by Iglesias-Puig and Haros [17] in bread with chia at 5% substitution. 

As compared to the control, chia flour incorporation increased the content of total dietary fiber by approximatively 2.3 times for Ch10-Lec0 and Ch10-Lec1blends and by 1.7 times for Ch5-Lec0.5 formulations. This tendency was also shown by other researchers who included chia flour in formulations of bread, spaghetti or cakes [18,19,20] and reported an increase in soluble dietary fiber content.

According to the World Health Organization (WHO), daily consumption of dietary fiber should be between 30–40 g. Its presence in the human diet helps to accelerate intestinal peristalsis, reduces the absorption of cholesterol and triglycerides, decreases glucose levels in the blood, and reduces the hunger sensation [21,22]. In this sense, the intake of 100 g of bread with 10% of chia could contribute by nearly 27.4% of this daily recommendation. In addition, together with higher doses of chia flour, there occurred an essential increase in the total content of fat and proteins has been noticed. The highest contents (12.13% of proteins and 3.11% of lipids) were found in the formulations involving the highest amount of chia flour (10%). The same tendency was observed with ash content which increased significantly with the addition of chia flour. For example, ash content from 2.04% in control bread to 2.23% in Ch10-Lec1 formulation (Table 3). 

### 3.3. Technological Quality of Fresh and Stored Bread Samples 

#### 3.3.1. Specific Volume

Fresh bread specific volume was determined one hour after taking it out from the oven. The different loaves specific volume ranged from 3.22−3.45 cm^3^/g. Compared to control, bread produced by the exclusive addition of lecithin at 1% presented a slight increase of the specific volume. This result may be explained by lecithin dough strengthening effect, which is not fully understood [23,24].

One theory suggests that lecithin potential to confer strength to wheat dough is due to the creation of a lecithin-gluten complex [25]. This emulsifier may bind to the hydrophobic surface promoting the aggregation of gluten proteins in dough. A strong protein network results in better texture and bread volume [25,26]. Another theory is based on lecithin ability to form liquid–crystalline phases in water, which associates with gliadin. These structures may contribute to dough elasticity allowing gas cell to expand, resulting in an increased volume of baked food [26].

Bread containing only chia flour at 10% showed a specific volume similar to the control sample despite gluten dilution. In contrast, Coelho & Salas-Mellado [27] showed that enriched bread with 11% of hydrated chia seeds had a significantly smaller specific volume than the control. In the mixed formulations, wheat flour gluten dilution by other flours that are free of this protein is responsible for starch-gluten matrix interruption and CO2 low retention during fermentation, resulting in a significant reduction of the specific volume [28,29]. However, in this study seeds were not pre-hydrated. Only chia flour was hydrated during the mixing step. This could result in forming a tridimensional network with protein and gluten molecules which compensated gluten dilution and consequently did not impair the specific volume, as compared to the control. 

The most important result to note is the positive effect of chia and lecithin combination on the specific volume. Regardless the added amounts, for the two formulations (Ch5-Lec0.5and Ch10-Lec1), the specific volume increased nearly to the same value 3.45 and 3.44 cm^3^/g. 

Therefore, we can conclude that lecithin incorporation not only corrected gluten proteins weakness caused by chia flour substitution, but also improved and strengthened these proteins (Table 3). 

#### 3.3.2. Crumb Moisture

A higher moisture content leads to a better quality of the crumb during storage, meaning a softer bread crumb. The moisture decrease affects the crumb firming rate since cross links will be created between partially solubilized starch and gluten proteins [5].

In the current study, moisture content in fresh bread crumbs decreased slightly with the following formulations Ch10-Lec0 > Ch0-Lec1 > Ch5-Lec0.5, in comparison with control fresh bread (43.7%) (Table 3). 

**Figure 1:**
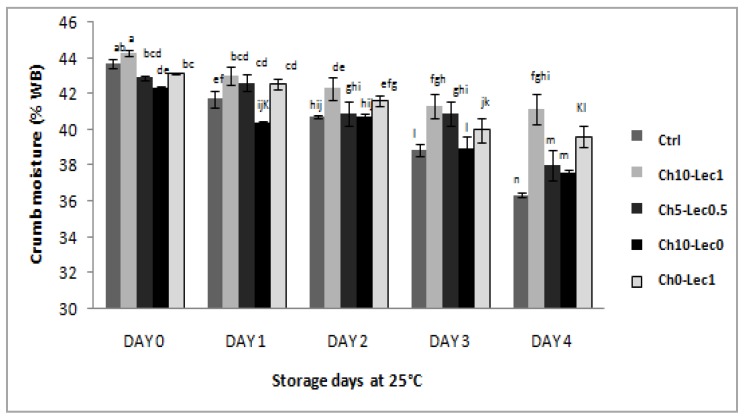
Crumb moisture of the different formulations during storage at 25 °C. WB: wet basis. a–h: Bars followed with the same letter are not significantly different at *p* < 0.05. Ch10-Lec0, bread with the exclusive addition of chia flour at 10%; Ch0-Lec1, bread with the exclusive addition of soy lecithin at 1%; Ch10-Lec1, bread with the addition of chia flour at 10% and soy lecithin at 1%; Ch5-Lec0.5, bread with the addition of chia flour at 5% and soy lecithin at 0.5%; ctrl, control bread without chia and lecithin.

Although the important ability of chia flour to absorb water during dough mixing, fresh bread crumbs presented lower moisture. This may be explained by the fact that the integrated chia flour absorbs water during dough formation and easily releases it during cooking [30]. However, from day 2 onward, reference bread moisture content became the lowest and it significantly decreased at day 4 (36.36%), as compared to the other bread samples which maintained a relatively higher humidity (between 37.56% and 41.17%) (Figure 1). 

Thus, the importance of chia mucilage water retention capacity appears during storage. The property of decreasing water loss in this phase of the process was also described by some studies. Likewise, Vázquez-Ovando et al. [31] reported that the fiber-rich fraction of defatted chia has a good water-holding capacity, besides its high antioxidant activity. 

From another side, compared to reference bread, only fresh breads of the formulation Ch10-Lec1 presented higher crumb moisture (44.3%) which maintained its level until the end of storage (41.17%). Thus, only the interaction of lecithin at 1% and chia at 10% resulted in a stronger water holding capacity during baking and storage. 

This result may be explained by a cooperative effect between the two components. Lecithin role began from the mixing step by the adsorption onto the starch surface which might not allow the starch granules to take up water released by gluten after baking to the same extent as the control bread [32]. Therefore, this water would be available for migration from the crumb to the crust during storage. Henceforth, the role of chia mucilage begins as a hydrocolloid with an important water retention ability resulting from its hydrophilic nature. In addition, the mucilage-gluten-starch network already formed during baking could act as a barrier to gas diffusion, decreasing thereby water vapor losses during storage.

#### 3.3.3. Crumb Texture 

Breads were daily submitted to texture analysis. Since consumers perceive bread with a softer crumb as more fresh, crumb firmness is one of the most critical texture parameters. At day 0, except bread sample containing only chia flour at 10%, which presented the highest crumb firmness (10.81 N), all the other bread samples baked either by a combination of chia and lecithin or by the exclusive incorporation of lecithin had a significantly lower firmness (between 4.36 and 5.6 N), as compared to reference bread crumb (7.47 N) (Table 3). These results demonstrated that crumb texture was affected by adding chia and lecithin to the formulation, in two different manners. 

On one hand, increasing crumb firmness by chia incorporation is mainly due to features related to dough gluten formation. Added flour dilutes the gluten matrix and causes crumb cell structure weakening. In fact, bread is a soft solid formed by two phases, a fluid one which corresponds to the air and a solid phase that corresponds to the gas-cell wall material. The solid phase is fully interconnected and the nature of the connectivity determines bread mechanical properties [3,4]. Consequently, a weaker gluten network develops a denser crumb, evidenced with a higher firmness.

On the other hand, Soy lecithin addition resulted in lower crumb firmness which can be explained by its capacity to improve air entrapment. A similar effect has also been proven for other bread emulsifiers such as the diacetyl tartaric acid esters of monoglycerides (DATEM) and the sodium stearoyl-2-lactylate (SSL) [26]. The interesting novelty is the effect of chia and lecithin combination on crumb firmness. Results showed that compared to crumb firmness of the bread containing only chia at a level of 10% (10.81 N), firmness value for bread produced with chia at 10% and lecithin at 1% decreased significantly (5.6 N). Similar effect was observed for enriched bread crumb with chia at 5% and lecithin at 0.5%, for which firmness value was 4.61 N (Table 3). We can thus conclude that there is a synergistic effect between chia and lecithin to decrease the initial bread crumb firmness. This emulsifier found in the same formulation with chia is supposed to correct and/or to promote the weakening effect of chia flour on gluten proteins, leading to softer fresh bread crumb.

**Figure 2:**
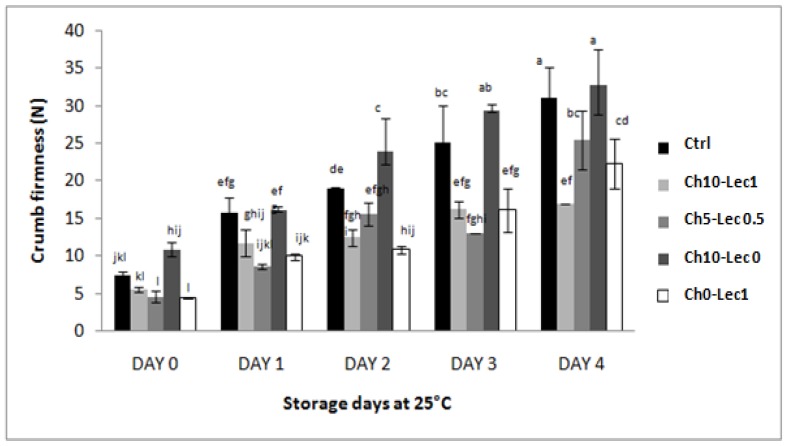
Crumb firmness of the different formulations during storage at 25 °C. N, Newton. a–l: Bars followed with the same letter are not significantly different at *p* < 0.05.

During storage, all bread crumbs’ firmness increased obviously as a result of staling. However, the same initial tendency was observed for all of them and the samples with improvers (except bread baked with 10% chia) maintained less firm crumbs. Also, it is interesting to note that, for Ch10-Lec1 sample, firmness increment was clear between day 0 and day 1, but henceforth crumb firmness increases in a non-significant way. At day 4, the highest values went to the control (30.95 N) and Ch10-Lec0 sample (32.79 N), with no statistical differences. Thus, despite gluten dilution at 10% of chia, final firmness of the correspondent bread crumb is statistically the same as the control bread. However, the sample with the exclusive addition of lecithin Ch0-Lec1 showed lower crumb firmness value (22.24 N), as compared to the reference. This value further decreased to reach 16.9 N in the sample containing chia and lecithin at maximal doses Ch10-Lec1 (Figure 2).

All these observations confirm the anti-staling effect of lecithin and the combination of lecithin and chia. Initially lecithin incorporation produced lower crumb hardness and a retarded the staling rate through its interaction with amylose, as well as with amylopectin. Lecithin—with a higher content of lysophospholipids—inhibits crumb firming, associated with staling by complexing with starch amylose [26].

The interactions between the lecithin and starch—adsorption process/complex formations— prevents starch from taking up water of the gluten network during the staling process [32]. Lecithin can also have an effect on moisture distribution between protein and starch fraction. A decrease in the amount of water absorbed by starch makes more water available for gluten hydration, contributing to staling delay.

From another side, knowing chia flour mucilage high water retention capacity, chia incorporation in the formulation Ch10-Lec0 was suspected to decrease water loss during storage and thus to retard staling. However, the contrasting results permit the conclusion that the diluting effect of chia flour on gluten network dominate water retention softening effect. This confirms the fact that lecithin-chia combination in the two formulations Ch10-Lec1 and Ch5-Lec0.5 allowed to correct the firming effect of chia. 

In summary, amphiphilic character of lecithin providing the possibility of forming complexes with starch, proteins, shortening and water [24], coupled to water retention capacity of chia mucilage permitted to ameliorate crumb texture and retard bread aging. 

Concerning the other textural attributes, except bread made with 10% chia for which crumb chewiness was the highest (6.46 N), regardless of the added doses, the study showed that the bread either with chia and lecithin or with exclusive lecithin addition requires less work during fragmentation (from 3.37 N to 4.18 N) as compared to the control (6.03 N). This decrease of crumb chewiness is favorably perceived by consumers.

**Figure A1:**
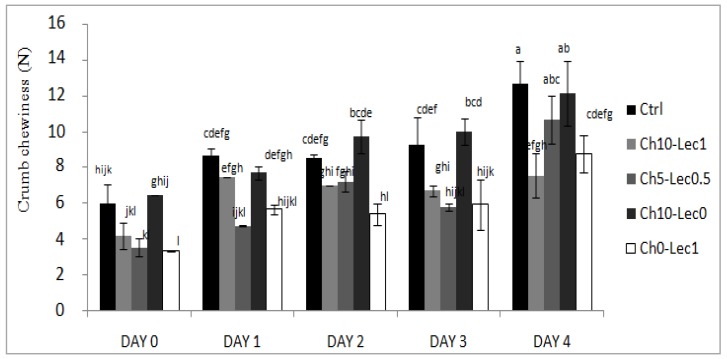
Crumb chewiness of the different formulations during storage at 25 °C.

**Figure A2:**
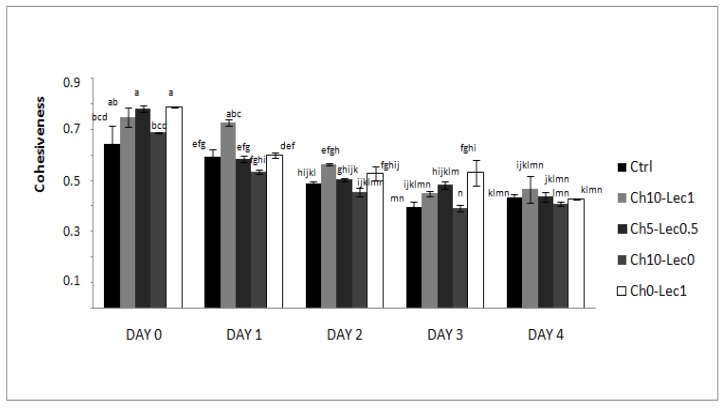
Crumb Cohesiveness of the different formulations during storage at 25 °C.

Cohesiveness of all enriched bread crumbs increased significantly up to 0.78. As reported by Onyango et al. [33], cohesiveness characterizes the extent to which a material can be deformed before it ruptures and reflects the internal cohesion of the crumb. The bread crumb with high cohesiveness is desirable because it forms a bolus instead of disintegrating during mastication whereas low cohesiveness indicates increased bread susceptibility to fracture or crumble. On the other hand, chia and lecithin addition had no significant influence on crumb springiness (Table 3). During storage, chewiness of the bread crumb samples increased whereas cohesiveness decreased significantly, while maintaining the same initial trend. Springiness is the only textural parameter which showed no significant difference between all fresh crumb samples and during their storage (Figure A1 and Figure A2). 

In general, bread textural properties (especially crumb firmness) are unfavorable for consumption at day 2 onward. However, our results confirm that fresh bread of Ch10-Lec1 formulation had the lowest crumb firmness which was maintained until the end of storage with no significant difference between day 2 and day 4. In contrast, control crumb firmness varied between 15.76 N on day 2 and 30.95 N on day 4.

**Figure 3:**
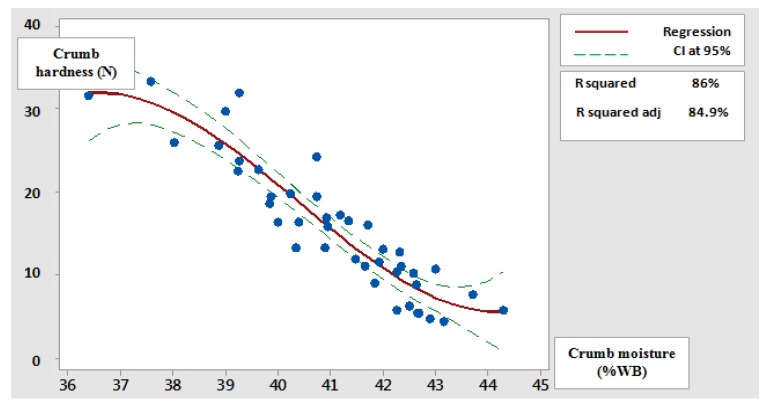
Adjustment of crumb firmness variation function of its moisture content. CI, Confidence Interval; R square adj., Adjusted R-Squared

To better understand and evaluate the interaction between crumb moisture content and its firmness, a cubic polynomial model was fitted to the experimental values. The adjustment trend confirmed the negative relation between the two variables. The more the crumb moisture increases, the more its firmness tends to decrease. This result was expected, seeing our results of moisture and firmness evolution mentioned above and knowing the plasticizing effect of water (Figure 3).

### 3.4. Modelling and Optimization of Blends Composition

#### 3.4.1. Models and Response Surfaces 

The obtained responses (Specific volume, crumb firmness day 0 and crumb firmness day 2) were statistically analyzed using the RSM, verifying the possibility of confirming the effects of chia and lecithin addition, mentioned previously, through mathematical models. Regression coefficients and variance analysis of the quadratic regression fitting models showing the relationships among responses and independent variables (chia and lecithin amounts) are presented in Table 4. R squared values which were higher than 80% reveals the good fit of the three models which explain 83.05%; 81.18% and 92.1% of the variation of the specific volume, crumb firmness day 0 and crumb firmness day 2, respectively.

Examining the interaction coefficients, it can be confirmed that the mutual influence of chia and lecithin to increase the specific volume of bread was significant. However, crumb firmness (day 0 and day 2) was negatively affected by this interaction, which was more significant at day 2 of storage (*p* < 0.01). This means that the effect of lecithin-chia interaction on reducing crumb firmness is more pronounced during storage than for fresh bread. 

**Figure 4:**
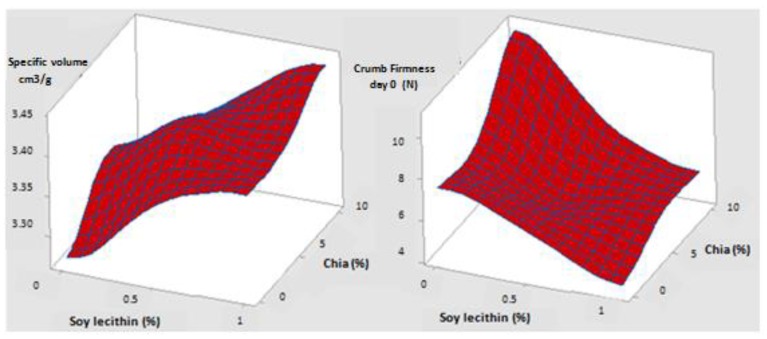
Response surfaces of crumb firmness (on the right) and specific volume (on the left) of fresh bread.

Observing Figure 4, it can be noted that, for fresh bread, with increasing doses of chia and lecithin, the general trend of the specific volume response surface was increasing whereas that of firmness tended to decrease. These two effects are desired to ameliorate the initial quality of fresh bread and to extend its shelf life. Another interesting clear result is that the desired effects are more pronounced at the maximal dose of lecithin (1%), as was expected.

**Figure 5:**
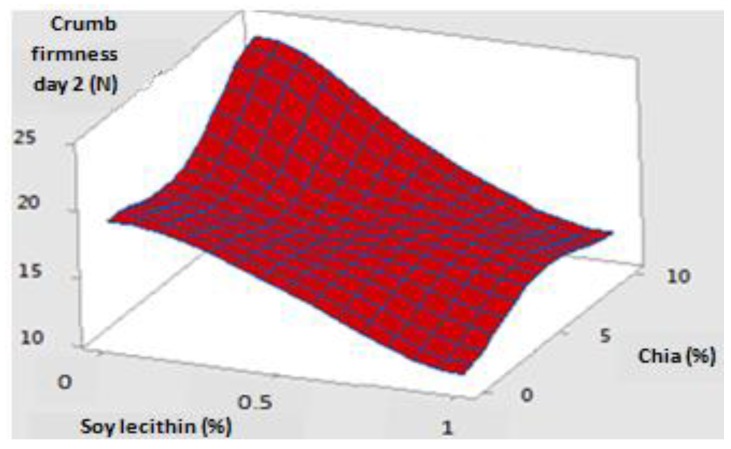
Response surface of crumb firmness of two days stored bread at 25 °C.

The same tendency was found for crumb firmness response surface after 2 days of storage at 25 °C, with only a displacement of the surface along the Z-axis, showing the increase in firmness during shelf life. Moreover, response surface of day 2 presented a plain with greater inclination, which reveals a greater effect of chia and lecithin interfering to retard crumb hardening as storage progresses (Figure 5).

#### 3.4.2. Optimization of Blends Composition

A numerical desirability function permitted to find out four blends of chia and lecithin in which the optimized doses were determined according four desired responses: maximum specific volume day 0; minimum firmness day 0; maximum specific volume and minimum firmness day 0; minimum firmness day 2.

**Figure 6:**
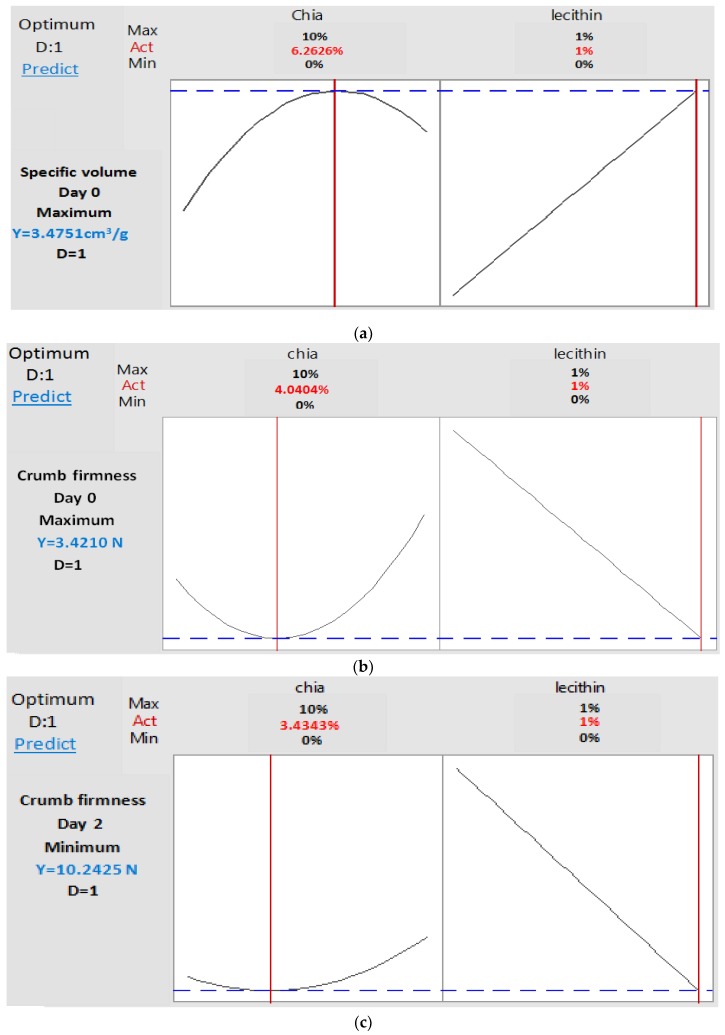
Optimized doses of chia and lecithin in order to maximize bread specific volume at day 0 (**a**), or minimize crumb firmness at day 0 (**b**) or minimize crumb firmness at day 2 (**c**). D, Desirability; Act, Action; Predict, prediction of the of the optimum response.

Through Figure 6, it can be concluded that, if the objective is to obtain fresh bread with maximum specific volume, the optimal blend is (6.26% chia; 1% lecithin) and the specific volume in this case will be equal to 3.47 cm^3^/g (Figure 6a). However, if we opt for bread with minimum crumb firmness equal to 3.24 N, the optimal mixture is (4.04% chia; 1% lecithin) (Figure 6b). 

**Figure 7:**
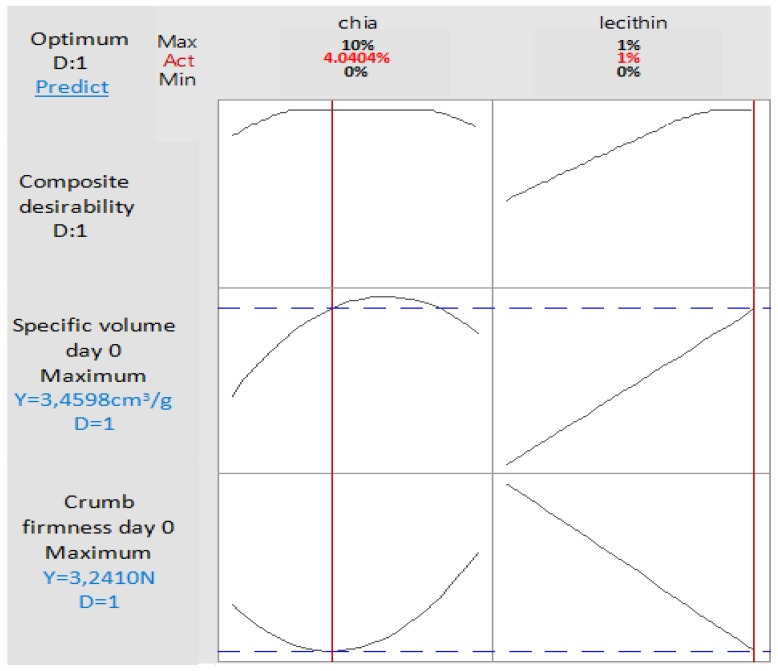
Optimized doses of chia and lecithin in order to maximize fresh bread specific volume and minimize its crumb firmness, simultaneously.

This mixture will be the same, if the objective is to obtain simultaneously fresh bread with minimum crumb firmness (3.24 N) and maximum specific volume (3.45 cm^3^/g) (Figure 7). 

Otherwise, if the aim is to retard bread staling and obtain bread with minimum crumb firmness (10.24 N) after two days of storage, the optimal blend should contain (3.43% Chia; 1% lecithin) (Figure 6c). Experimental values were close to the predicted ones, with an error below 10% for all tested attributes.

## 4. Conclusions 

The current study aimed to present that in addition to the functional and nutritional improvement of chia-added bread, an enhancement of fresh bread technological quality and a better resistance to staling during storage at 25 °C, was observed.

In fact, incorporating chia flour at 10% decreased bread flour specific volume, whereas, bread produced by lecithin and Chia addition was characterized with an increased specific volume. Also, only chia and lecithin combination resulted in a stronger water holding capacity during baking. Besides, crumb firmness increased with chia addition and decreased with lecithin incorporation. 

However, there is a synergistic effect between the two additives to decrease crumb firmness. Another fact which should be considered is the good effect on crumb textural attributes. A significant enhancement of cohesiveness and a reduction of chewiness of all enriched bread crumbs were observed.

During storage, regardless the added amounts, lecithin and chia combination resulted in a significant water loss and crumb firmness decrease, compared to samples containing only lecithin or only chia. This result confirms the synergy to increase water holding capacity and to delay bread staling.

As a perspective, the optimum blends of chia and lecithin may be used to make bread at different heating rates in order to study the effect of baking conditions on bread staling and to optimize the temperature and the time of cooking. Also, our results may be tested in another baking technology. For example, it will be interesting to study the effect of the optimal composition on the staling rate of part-baked bread.

## Figures and Tables

**Table 1:** Experimental design.

Assay	Codes	Chia (%)	Lecithin (%)
1	Ch5-Lec0.5	5 (0)	0.5 (0)
2	Ch5-Lec0.5	5 (0)	0.5 (0)
3	Ch5-Lec0.5	5 (0)	0.5 (0)
4	Ch5-Lec0.5	5 (0)	0.5 (0)
5	Ch10-Lec1	10 (1)	1 (1)
6	Ctrl	0 (−1)	0 (−1)
7	Ch5-Lec0.5	5 (0)	0.5 (0)
8	Ch0-Lec1	0 (−1)	1 (1)
9	Ch10-Lec0	10 (1)	0 (−1)

Ch10-Lec0, bread with the exclusive addition of chia flour at 10%; Ch0-Lec1, bread with the exclusive addition of soy lecithin at 1%; Ch10-Lec1, bread with the addition of chia flour at 10% and soy lecithin at 1%; Ch5-Lec0.5, bread with the addition of chia flour at 5% and soy lecithin at 0.5%; ctrl, control bread without chia and lecithin.

**Table 2:** Dough mixing properties of the different formulations.

Parameters	Units	Ctrl	Ch-Lec0.5	Ch10-Lec0	Ch0-Lec1	Ch10-Lec1
Absorption	%	57.2 ± 0.2 ^a^	59.5 ± 0.2 ^ab^	65.0 ± 0.4 ^c^	58.5 ± 0.3 ^ab^	65.0 ± 0.2 ^c^
Arrival time	min	1.0 ± 0.1 ^a^	1.2 ± 0.1 ^a^	1.4 ± 0.2 ^a^	1.3 ± 0.1 ^a^	1.3 ± 0.2 ^a^
Development time	min	1.9 ± 0.3 ^a^	2.5 ± 0.2 ^ab^	5.2 ± 0.1 ^b^	2.1 ± 0.1 ^a^	3.9 ± 0.3 ^b^
Stability	min	3.0 ± 0.2 ^a^	7.8 ± 0.4 ^b^	8.5 ± 0.3 ^b^	3.2 ± 0.2 ^a^	8.0 ± 0.2 ^b^
Break down time	min	4.0 ± 0.1 ^a^	9.0 ± 0.3 ^b^	9.9 ± 0.2 ^b^	4.5 ± 0.4 ^a^	9.3 ± 0.3 ^b^
Mixing tolerance index	BU	60.0 ± 0.5 ^b^	60.5 ± 0.4 ^b^	62.2 ± 0.4 ^c^	58.7 ± 0.5 ^a^	62.0 ± 0.3 ^c^

^a–d^ Means within lines followed by the same letter are not significantly different at 95% confidence level. Ch10-Lec0, bread with the exclusive addition of chia flour at 10%; Ch0-Lec1, bread with the exclusive addition of soy lecithin at 1%; Ch10-Lec1, bread with the addition of chia flour at 10% and soy lecithin at 1%; Ch5-Lec0,5, bread with the addition of chia flour at 5% and soy lecithin at 0.5%; ctrl, control bread without chia and lecithin.

**Table 3:** Proximal composition and technological parameters of fresh bread samples.

Parameter	Units	Ctrl	Ch10-Lec1	Ch5-Lec0.5	Ch10-Lec0	Ch0-Lec1
Proximal Composition						
Proteins	%, d.m.	11.0 ± 0.3 ^a^	12.1 ± 0.3 ^c^	11.7 ± 0.1 ^b^	12.1 ± 0.4 ^c^	10.9 ± 0.3 ^a^
Lipids	%, d.m.	1.1 ± 0.5 ^a^	3.1 ± 0.3 ^b^	2.8 ± 0.7 ^c^	2.9 ± 0.5 ^cd^	1.2 ± 0.4 ^a^
Total Dietary Fibers	%, d.m.	3.6 ± 0.1 ^a^	8.2 ± 0.4 ^b^	6.1 ± 0.2 ^c^	8.2 ± 0.3 ^b^	3.6 ± 0.1 ^a^
Ash content	%, d.m.	2.04 ± 0.01 ^a^	2.23 ± 0.01 ^b^	2.15 ± 0.01 ^b^	2.21 ± 0.01 ^b^	2.07 ± 0.01 ^a^
Total moisture	%	35.90 ± 0.03 ^e^	35.00 ± 0.00 ^d^	34.00 ± 0.04 ^c^	32.90 ± 0.00 ^a^	33.20 ± 0.01 ^b^
Technological parameters						
Volume	cm^3^	281 ± 1 ^a^	289.3 ± 0.5 ^b^	288 ± 1 ^b^	280.9 ± 0.9 ^a^	287 ± 1 ^b^
Specific volume	cm^3^/g	3.27 ± 0.008 ^b^	3.45 ± 0.03 ^d^	3.44 ± 0.01 ^d^	3.22 ± 0.01 ^a^	3.30 ± 0.04 ^c^
Crumb moisture	%	43.70 ± 0.2 ^c^	44.30 ± 0.09 ^d^	42.9 ± 0.1 ^b^	42.30 ± 0.04 ^a^	43.10 ± 0.07 ^b^
*Crumb texture*						
Firmness	N	7.5 ± 0.4 ^b^	5.6± 0.3 ^ab^	4.6± 0.7 ^a^	10.8 ± 1.0 ^c^	4.36 ± 0.04 ^a^
Cohesiveness		0.64 ± 0.17 ^a^	0.74 ± 0.03 ^b^	0.78 ± 0.01 ^b^	0.68 ± 0.00 ^b^	0.78 ± 0.00 ^b^
Springiness		1.09 ± 0.49 ^a^	0.97 ± 0.07 ^a^	0.97 ± 0.00 ^a^	0.92 ± 0.00 ^a^	0.96 ± 0.01 ^a^
Chewiness	N	6.03 ± 0.5 ^bc^	4.18 ± 0.3 ^abc^	3.56 ± 0.3 ^ab^	6.46 ± 0.0 ^c^	3.37 ± 0.2 ^a^

Results are expressed as (x ± d), being x the average and d, the standard deviation, (*n* = 3). ^a–d^ Means within lines followed by the same letter are not significantly different at 95% confidence level. N, Newton; d.m. dry matter

**Table 4:** Regression coefficients and analysis of variance of the quadratic regression fitting models.

Factor	Specific Volume Day 0	Crumb Firmness Day 0	Crumb Firmness Day 2
Constant	3.27	7.47	19.05
Chia	0.0246 *	−0.339 *	−0.048 *
lecithin	0.12ns	−3.11 **	−8.19 **
Chia x lecithin	0.041 *	−0.21 *	−0.313 **
Chia^2^	−0.00206 **	0.0673 *	0.0528 *
Lecithin ^2^	0	0	0
R^2^	84.97	84.54	96.05
Adjusted R^2^	83.05	81.18	92.11

* *p* ≤ 0.05; **, *p* ≤ 0.01; ns, *p* > 0.05. R2 ≥ 80 indicates a good fit to the polynomial equations.
